# Accentuating the detection of immunological signatures as a diagnostic tool for monkeypox virus

**DOI:** 10.1097/JS9.0000000000000131

**Published:** 2023-03-03

**Authors:** Vishnu Priya Veeraraghavan, Lavina Prashar, Sreenidhi Prakash, Jyotsna Needamangalam Balaji, Ullas Mony, Krishna Mohan Surapaneni

**Affiliations:** a Centre of Molecular Medicine and Diagnostics (COMManD), Saveetha Dental College and Hospitals, Saveetha Institute of Medical and Technical Sciences, Saveetha University; b Panimalar Medical College Hospital & Research Institute; c SMAART Population Health Informatics Intervention Center, Foundation of Healthcare Technologies Society, Panimalar Medical College Hospital & Research Institute; Departments of d Biochemistry; e Medical Education; f Molecular Virology; g Research; h Clinical Skills & Simulation, Panimalar Medical College Hospital & Research Institute, Chennai, Tamil Nadu, India; i Department of Physiological Sciences, School of Medicine, Unza Ridgeway Campus, University of Zambia, Lusaka, Zambia

*Dear Editor*,

The WHO declared the recent monkeypox surge as a Public Health Emergency of International Concern on 23 July 2022. Rapid measures are being taken in terms of diagnosis, immunization, treatment and management of the disease to prevent another catastrophe. Although monkeypox disease is not proven to be fatal for majority of the people, early diagnosis and appropriate therapeutic regiment has to be followed to curb the transmission of virus. Currently, the predominant diagnostic test performed is the Centers for Disease Control and Prevention (CDC)’s Nonvariola-Orthopox Virus PCR test using samples of skin lesions^1^. Such invasive methods pose the risk of infection, contamination and reluctant behaviour from patients and healthcare staff while obtaining the sample. This raises the need for a simple, feasible yet accurate mode of detection such as blood or other body fluid investigations. But there is lack of evidence on the use of blood, saliva or other body secretions for detection and confirmation of virus. This article explores the available literature on the detection of immunological signatures in blood as a probable testing method for diagnosing monkeypox virus.

The altered kinetics in antiviral antibody response is evident among the confirmed cases of monkeypox disease indicating a significant association between onset of rashes and pattern of rise of immunoglobulins (Igs) in patients’ sera. The viral culture results and PCR finding were found to be were consistent with the seroconversion of Igs. IgM enzyme-linked immunosorbent assay and IgG enzyme-linked immunosorbent assay were thus efficient as diagnostic indicators and phase-of-illness indicators of monkeypox infection^2^. In addition to this, comparing the serum signatures between monkeypox infected monkey and human cell lines, a characteristic elevation of cluster of differentiation (CD) 40, plasmin and histamine was seen in monkey cell lines, while interferons macrophages and neutrophil-related signalling pathways were predominant in human cell lines^3^. However, these findings have not been studied in a larger cohort and hence require more intense clinical research to establish these biological constituents as immunological signatures of monkeypox disease.

Monkeypox disease has also been strongly associated with early T-cell response and increased inflammatory mediators. An early elevation of CD8^+^ T cells and reduction in CD4^+^ T cells is seen in patients with monkeypox virus. This is combined with high expression of CD38 activation marker. This elevated T-cell pattern was found to be normalized within 12–20 days from the onset of symptoms. Inflammatory markers such as interleukins, particularly IL-6, IL-8, IL-16 and tumour necrosis factor are also found to be elevated in these patients. A specific characteristic finding was the detection of Th-1 biased pox-specific T cells in the serum. This antigen specific finding was almost predominant in all of the patients diagnosed with monkeypox virus^4^,^5^.

Although these studies have provided information about the use of immune markers in detection of monkeypox, the laboratory method of using these immunological signatures for diagnosing monkeypox and disease progression is still at its nascent stage. The detection of immune markers in patient’s serum to identify monkeypox disease will improve the quality and efficacy of testing when compared with conventional skin lesion sampling. Thus, we emphasize on conducting more clinical trials and evidence-based studies to make such immune signatures reliable and accurate for disease detection.

## Ethical approval

Not applicable.

## Sources of funding

None.

## Authors’ contribution

V.P.V.: conceptualization, data curation, writing – original draft preparation. L.P., S.P., J.N.B., U.M.: data curation, writing – original draft preparation. K.M.S.: conceptualization, study design, data curation, writing – original draft preparation, reviewing and editing, visualization and supervision.

## Conflicts of interest disclosure

The authors declare that they have no financial conflict of interest with regard to the content of this report.

## Research registration unique identifying number (UIN)

Not applicable.

## Guarantor

Krishna Mohan Surapaneni.
